# Trends in U.S. National Institutes of Health Funding for CHARGE Syndrome Research, 2000 to 2024

**DOI:** 10.1002/ajmg.a.64201

**Published:** 2025-08-07

**Authors:** Muhammad Othman, Lillian J. Slavin, Tyler G. James

**Affiliations:** 1 Department of Family Medicine, University of Michigan, Ann Arbor, Michigan, USA; 2 Swank Autism Center, Nemours Children’s Health, Wilmington, Delaware, USA; 3 Center for Social Epidemiology and Population Health, Department of Epidemiology, University of Michigan, Ann Arbor, Michigan, USA; 4 Center for Bioethics and Social Sciences in Medicine, University of Michigan, Ann Arbor, Michigan, USA

**Keywords:** CHARGE syndrome, deafblindness, National Institutes of Health, research funding

CHARGE syndrome (OMIM 214880) is a rare and complex genetic disorder causing a variety of manifestations across body systems, including coloboma, congenital heart defects, deafness, and cranial nerve differences (Thomas et al. 2022; van Ravenswaaij-Arts et al. 1993). The incidence of CHARGE syndrome is estimated to be between 1-in-15,000 and 1-in-8500 (Issekutz et al. 2005; Janssen et al. 2012). Despite its rarity, it is one of the leading causes of congenital DeafBlindness in the United States (National Center on DeafBlindness 2023). Almost all people with CHARGE syndrome have a developmental delay/disability (Moss and Howlin 2009; Thomas et al. 2022; van Ravenswaaij-Arts et al. 1993). Over 90% of patients who meet the clinical diagnostic criteria of CHARGE syndrome have a mutation in the CHD7 gene (Bergman et al. 2011; Usman and Sur 2024), with some studies reporting as low as 70% prevalence of pathogenic CHD7 variants (Jongmans et al. 2006). CHARGE presents unique challenges in diagnosis, management, and prognosis, affecting both the quality and duration of life for individuals and their families. Despite its significant clinical and social implications, CHARGE syndrome remains underrepresented in medical research due to its rarity.

The National Institutes of Health (NIH) in the United States is the largest biomedical research funder in the world, and NIH support has been essential for advancing understanding, developing interventions, and improving outcomes for rare diseases like CHARGE syndrome (Brooks et al. 2024; Kaufmann et al. 2018). Analyzing funding trends allows researchers, clinicians, and policymakers to assess how resources are allocated and identify gaps and opportunities to increase innovation in specific areas of study. For example, similar analyses conducted in autism spectrum disorder and other rare conditions have illuminated disparities in funding allocation and have informed decision-making for research priorities (Cervantes et al. 2021; Fagnan et al. 2015).

To date, the extent of NIH funding dedicated to CHARGE syndrome-related research has not been systematically analyzed. Understanding these trends can provide critical insight into the NIH’s research priorities and inform strategies for addressing unmet needs in this population. The objective of this study was to assess the NIH’s investment in CHARGE syndrome research from 2000 to 2024, with a focus on identifying funding trends, associated NIH institutes, and translational research stages.

We conducted a retrospective analysis of research projects funded by the NIH related to CHARGE syndrome using the NIH Research Portfolio Online Reporting Tools Expenditures and Results (RePORTER) database. This publicly accessible database provides detailed information on NIH-supported projects, including titles, abstracts, funding amounts, and the associated funding institute or center. To identify relevant projects, we performed a keyword-based search using the search terms and associated Medical Subject Headings for “CHARGE syndrome”, “CHD7”, and “CHARGE association” in project titles and abstracts. The search covered the period from January 1, 2000, to December 31, 2024.

The exported data file from NIH RePORTER was transferred into REDCap for review.

Each identified project was manually reviewed by two reviewers (M.O. and T.G.J.) to assess its relevance to CHARGE syndrome, based on explicit references to CHARGE syndrome or CHD7-related disorders in the project objectives, methodologies, or outcomes. Disagreements were arbitrated through discussion with a third expert (L.S.). Data points extracted from the RePORTER database included total funding amount in U.S. dollars ($), the NIH institute or center providing the funding, and detailed project descriptions, including titles, abstract narratives, and public health relevance statements. To better understand the focus of research activities, each project was categorized into one of five translational research stages (T0–T4); these stages identify the type of research, from basic, pre-clinical, and animal study (T0) to implementation, public health impact studies (T4). We used this classification to evaluate the balance between fundamental research and applied studies and help identify patient-centered research.

Data were analyzed descriptively to provide an overview of NIH funding trends in CHARGE syndrome-related research over the 24-year period. We calculated the total number of funded projects and the cumulative funding amounts and examined how these were distributed across NIH institutes/centers. Additionally, the distribution of projects across translational research stages was assessed to determine the focus on basic versus applied research.

In total, 45 unique projects were funded between 2000 and 2024 (Figure 1). These projects represented a total of $64.3 million USD in NIH funding (Table 1). Most of the support was from the National Eye Institute, followed by the National Institute on Deafness and Other Communication Disorders. The independent large-scale research project mechanism, known as an R01, was the most frequent and largest grant type across all years. Finally, most of the research in CHARGE syndrome is invested at the T0 basic research stage, with only $3000 invested outside of the T0 stage. A detailed table with all categories across funding institutes/centers and activity codes is available in Table S1.

To our knowledge, this is the first formal funding analysis of CHARGE syndrome-related research. Our findings indicate that the U.S. NIH invested $64 million in total costs for CHARGE syndrome-related research. This investment is relatively lower than that of other rare diseases. For example, between 2008 and 2023, Usher syndrome—another rare genetic disease causing DeafBlindness—had an NIH investment of $175 million; this was 3 times more funding than CHARGE syndrome in the same time period (National Institutes of Health 2024). Other rare neurodevelopmental disabilities, like Rett syndrome (National Institutes of Health 2003) and Fragile X (Trans-NIH Fragile X Coordinating Committee 2019), have received significant investment from the NIH. This investment is not limited to the NIH; Rett syndrome, for example, a condition rarer than CHARGE syndrome—is specifically eligible for funding from the U.S. Department of Defense Congressionally Directed Medical Research Program. Members of Congress are able to request condition inclusion in the Congressionally Directed Medical Research Program (Mendez 2025); it is possible that rare disease organizations, focused on conditions eligible through this mechanism, have conducted significant advocacy with Congressional leaders for funding.

Over the past 24 years, investment in CHARGE syndrome-related research from NIH has grown. This is likely due to the identification of a causal gene (CHD7) in 2004 (Vissers et al. 2004) The investments in CHARGE syndrome have been from institutes/centers that are related to major manifestations present in CHARGE syndrome: coloboma (National Eye Institute), deafness (National Institute on Deafness and Other Communication Disorders), and general child development (National Institute on Child Health and Human Development). Most insightful, however, is that essentially all of the NIH funding on CHARGE syndrome has been at the basic research (T0) stage.

Overall, the lack of research outside of the T0 stage indicates a need to ensure appropriate translation of findings to later stages and, ultimately, the need to ensure research is benefiting patients and families. Compared to other rare diseases that have received relatively more investment from federal funders, the clinical trial readiness and conduct of patient-centered clinical research in CHARGE syndrome need to be improved. This will require a multi-pronged approach: (1) expansion of funding opportunities, (2) delineation of research priorities, (3) improvement in recruitment and retention, and (4) workforce development.

First, the Department of Defense’s Congressionally Directed Medical Research Program—Peer-Reviewed Medical program provides support to research on select rare diseases. CHARGE syndrome has not been listed as a rare disease eligible for this program. Therefore, we recommend advocacy to encourage Congress to add CHARGE syndrome as an eligible condition. The second recommendation requires action from patients, families, advocacy organizations, researchers, and federal funders: delineate the research priorities and establish a research agenda for CHARGE syndrome. Defining a research agenda will help ensure that research is aligned with patient and family needs, is focused on developing a pipeline of research that will eventually lead to improved care coordination and therapeutics (with the goal of improving the quality-of-life of people with CHARGE syndrome) and can be used as a tool to advocate for increased research funding. Recruitment remains a challenge in rare disease translational research—CHARGE syndrome is no exception. We recommend the development of research networks that can aid in pooling data across multiple clinical sites. Lastly, work-force development programs will help ensure there are clinical and translational scientists who are well positioned to apply for expanded funding opportunities. We support continuation and expansion of workforce development in CHARGE syndrome and related disabilities. These programs include The CHARGE Syndrome Foundation’s Davenport Fellowship, the Rare Disease Clinical Research Training Program supported by the NIH, and the Leadership Education in Neurodevelopmental and Other Related Disabilities (LEND) training supported by the Health Resources and Services Administration.

The data source for this study was limited to narrative and public health summary statements, not the full grant application text. Therefore, it is possible that there was a misclassification of grant awards included in this study. For example, some projects had implications for neural crest cell development; changes in neural crest cell development are one of the hypothesized mechanisms of CHD7 mutations causing CHARGE syndrome. Similarly, some projects focused specifically on CHD7 but not aspects related to CHARGE syndrome; for example, one project focused on the role of CHD7 in colorectal cancer. Although this study was not specific to CHARGE syndrome, the focus on CHD7 mechanisms, protein interactions, and epigenetic regulation could be useful in CHARGE syndrome-related projects. (The contributions of CHD7-related research across multiple fields likely also contributes to the amount of T0 funding identified in this study.) Lastly, we focused on funding from the U.S. NIH and not from foundation or intramural funders.

In conclusion, the U.S. National Institutes of Health has funded $64 million in total costs for CHARGE syndrome-related research between 2000 and 2024. Almost all this funding has been basic biomedical research, without direct translation to patient care. We recommend expanding funding opportunities and the field of CHARGE syndrome research, defining strategic research priorities to achieve clinical trial readiness and improve patients’ lives.

## Supplementary Material

supplement

Additional supporting information can be found online in the Supporting Information section. **Table S1:** Characteristics of grants awarded by the U.S. National Institutes of Health (NIH) for CHARGE syndrome-related research between 2000–2020.

## Figures and Tables

**FIGURE 1 |:**
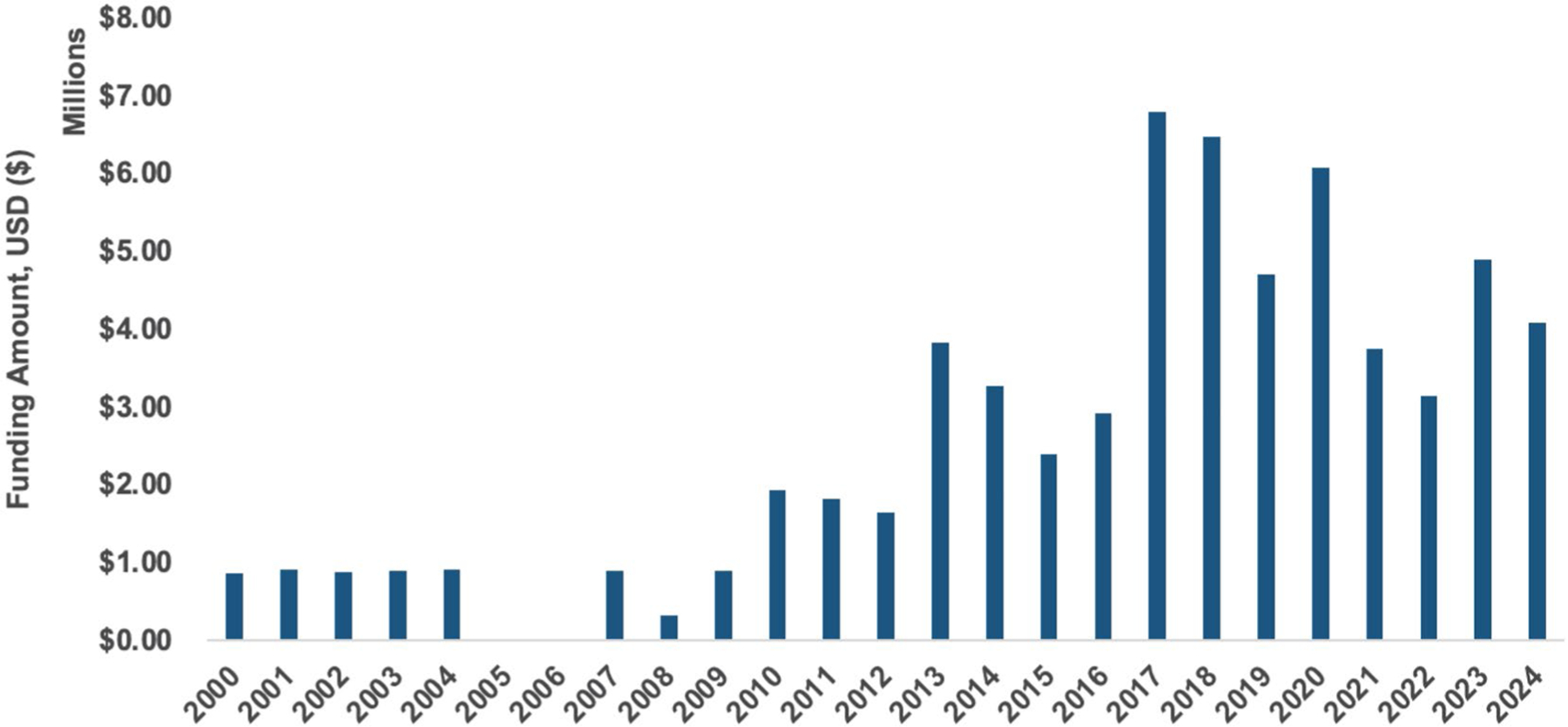
Annual amount of funding for CHARGE syndrome-related research by the U.S. National Institutes of Health between 2000 and 2024, in millions of U.S. dollars.

**TABLE 1 |:** Summary of research studies funded by the United States National Institutes of Health on CHARGE syndrome between 2000 and 2024.

Characteristic	Number of projects	Funding amount ($, USD)
All projects	45	$64,285,862
Institute/center		
National Eye Institute	3	$12,896,496
National Institute on Deafness and Other Communication Disorders	9	$11,647,840
National Institute on General Medical Sciences	4	$9,189,349
*Eunice Kennedy Shriver* National Institute on Child Health and Human Development	5	$8,901,662
Other	24	$21,650,515
Stage of translational research		
T0: Basic research to define the mechanisms of health or disease (e.g., animal models)	44	$64,282,862
T1: Applying understand of mechanisms to health of humans (e.g., proof of concept, biomarker studies)	—	—
T2: Developing evidence-based practice (e.g., Phase 1, Phase 2 clinical trials)	—	—
T3: Comparing to widely adopted health practice (e.g., comparative effectiveness, pragmatic studies)	—	—
T4: Improving population or community health by optimizing interventions (e.g., cost-effectiveness, policy or environmental change, population epidemiology)	1	$3000
Activity code/mechanism		
R01: Research project	21	$38,333,973
ZIA: Investigator initiated intramural research projects	2	$12,935,522
R35: Outstanding investigator award	1	$4,280,029
R21: Exploratory/developmental grants	7	$2,934,328
R37: Method to extend research in time (MERIT) award	1	$1,979,250
P50: Specialized center	1	$1,160,839
Other	12	$2,662,001

## Data Availability

The data that support the findings of this study are available in NIH Research Portfolio Online Reporting Tools Expenditures and Results (RePORTER), a publicly accessible database. The curated dataset for this study will be available at DOI: 10.6084/m9.figshare.28551986 after July 1, 2025.
