# Supplementary material for: Trends in U.S. National Institutes of Health Funding for CHARGE Syndrome Research, 2000 to 2024
Source: Am J Med Genet A. Author manuscript; Available in PMC 2026 Feb 9. (PMC12884510; doi:10.1002/ajmg.a.64201)
Supplement: supplement [file NIHMS2137432-supplement-supplement.docx]

**Supplemental Table 1.** Characteristics of grants awarded by the U.S. National Institutes of Health (NIH) for CHARGE syndrome-related research between 2000-2020.

| **Characteristic** | **Number of Projects** | **Funding Amount ($, USD)** |
| --- | --- | --- |
| All projects | 45 | $64,285,862 |
| Funder |  |  |
| National Eye Institute | 3 | $12,896,496 |
| National Institute on Deafness and Other Communication Disorders | 9 | $11,647,840 |
| National Institute on General Medical Sciences | 4 | $9,189,349 |
| *Eunice Kennedy Shriver* National Institute on Child Health and Human Development | 5 | $8,901,662 |
| National Heart, Lung, and Blood Institute | 4 | $4,675,947 |
| National Institute of Neurological Disorders and Stroke | 6 | $4,640,578 |
| National Institute of Dental and Craniofacial Research | 4 | $2,908,293 |
| National Cancer Institute | 2 | $2,134,101 |
| Office of the Director | 1 | $2,039,419 |
| National Human Genome Research Institute | 1 | $1,524,807 |
| National Institute of Mental Health | 2 | $1,452,658 |
| National Institute of Allergy and Infectious Diseases | 2 | $1,293,797 |
| National Institute on Aging | 1 | $560,327 |
| National Center for Research Resources ^a^ | 1 | $420,588 |
| Stage of translational research |  |  |
| T0: Basic research to define the mechanisms of health or disease (e.g., animal models) | 44 | $64,282,862 |
| T1: Applying understand of mechanisms to health of humans (e.g., proof of concept, biomarker studies) | - | - |
| T2: Developing evidence-based practice (e.g., Phase 1, Phase 2 clinical trials) | - | - |
| T3: Comparing to widely adopted health practice (e.g., comparative effectiveness, pragmatic studies) | - | - |
| T4: Improving population or community health by optimizing interventions (e.g., cost-effectiveness, policy or environmental change, population epidemiology) | 1 | $3,000 |
| Activity code/mechanism ^b^ |  |  |
| R01: Research project | 21 | $38,333,973 |
| ZIA: Investigator initiated intramural research projects | 2 | $12,935,522 |
| R35: Outstanding investigator award | 1 | $4,280,029 |
| R21: Exploratory/developmental grants | 7 | $2,934,328 |
| R37: Method to extend research in time (MERIT) award | 1 | $1,979,250 |
| P50: Specialized center | 1 | $1,160,839 |
| R00: Research transition award | 1 | $628,748 |
| Z01: Intramural research project | 1 | $560,327 |
| P01: Research program project | 1 | $380,491 |
| R56: High priority, short term project award | 1 | $367,500 |
| F30: Individual predoctoral National Research Service Award for MD/PhD fellowships | 2 | $268,814 |
| F32: Postdoctoral individual National Research Service Award | 1 | $167,646 |
| F31: Predoctoral individual National Research Service Award | 3 | $143,787 |
| R03: Small research grant | 1 | $141,698 |
| R13: Conference | 1 | $3,000 |

^a^ The National Center on Research Resources (NCRR) was established in 1990 and was dissolved in 2011 as part of a reorganization into the National Center for Advancing Translational Sciences (NCATS).

^b^ Intramural research awards are those that are taking place by investigators who are employed by the NIH. Common research grant awards are R01, R03, and R21. Individual training awards include predoctoral (F30, F31), postdoctoral (F32), and career development awards (R00, K-mechanisms). A full description of activity codes and mechanisms are at <https://grants.nih.gov/funding/activity-codes>.
